# Osteomyelitis of Maxilla in Infantile Osteopetrosis: A Case Report with Review of Literature

**DOI:** 10.5005/jp-journals-10005-1095

**Published:** 2010-04-15

**Authors:** Anita Balan, KL Girija, P Ranimol

**Affiliations:** 1 Professor and Head, Department of Oral Medicine and Radiology, Government Dental College, Thiruvananthapuram, Kerala, India; 2 Lecturer, Department of Oral Medicine and Radiology, Government Dental College, Thiruvananthapuram, Kerala, India; 3 Postgraduate Student, Department of Oral Medicine and Radiology, Government Dental College, Thiruvananthapuram, Kerala, India

**Keywords:** Osteomyelitis, Osteopetrosis.

## Abstract

Osteopetrosis is a rare genetic disorder that causes generalized sclerosis of bone due to a defect in bone resorption and remodeling. It is usually manifesting in two basic forms: An autosomal dominant benign form (osteopetrosis tarda) and an autosomal recessive malignant form (osteopetrosis congenita). A third form, the intermediate recessive type, has also been reported. Dental abnormality may be attributed to pathological changes in bone remodeling. Osteomyelitis is well documented as a complication of osteopetrosis and is severe and difficult to treat. This is a case of 8-year-old boy with osteopetrosis presenting with the complaint of swelling of left side of face.

## INTRODUCTION

Osteopetrosis (osteo means bone; petros means stone) is a rare hereditary generalized disorder of bone, characterized by a significant increase in the density of the skeletal tissues due to defect in bone resorption (osteoclastic activity) and remodeling. It is also known as marble bone disease or Albers-Schonberg disease. It is usually manifesting in two basic forms: An autosomal dominant benign form (osteopetrosis tarda) and an autosomal recessive malignant form (osteopetrosis congenita). A third form, the intermediate recessive type, has also been reported. In all three forms, pathologic alteration of osteoclastic bone resorption, thickening of cortical and lamellar bones are the main features.^1,2^ The incidence of OP is thought to be one in 100,000 to one in 500,000. OP exhibits a vast spectrum of clinical, physiologic and genotypic expressions.^3,4^ Patients with infantile malignant osteopetrosis are the most severely affected and even though more common than the intermediate recessive form, they are still rarely seen in the general population. Osteomyelitis secondary to odontogenic infection is a common complication in these patients.^4,5,8^ The disease can be severe and difficult to treat in these patients with resultant gross disfigurement due to surgical removal of the affected facial and skeletal bones.^3^ Thus, preventive measures directed at aborting caries and its sequelae or the need for extraction is recommended because they can be lifesaving for these patients.^4^ The purpose of this article is to present a case of OP with osteomyelitis of mandible and comprehensive review of its classification, pathophysiology and diagnosis. The importance of routine radiography is highlighted with a need for prompt care emphasized to avoid complications that might result in significant mortality in such patients.

## CASE REPORT

An 8-year-old boy reported to the outpatient clinic of Oral Medicine and Radiology, Government Dental College, Thiruvananthapuram, with complaints of painful swelling of left side of face of 4 days duration. He had been referred from the Department of Pediatrics, SAT Hospital, Thiruva-nanthapuram. There was history of hydrocephalus, delayed milestones of development (like delayed speech, walking, etc.). And recurrent hospitalization for treatment for severe dyspnea and anemia. There was history of fracture of left thigh two years back. A family history failed to reveal any significant findings among his parents or siblings. He had no hearing defect.

**Fig. 1:**
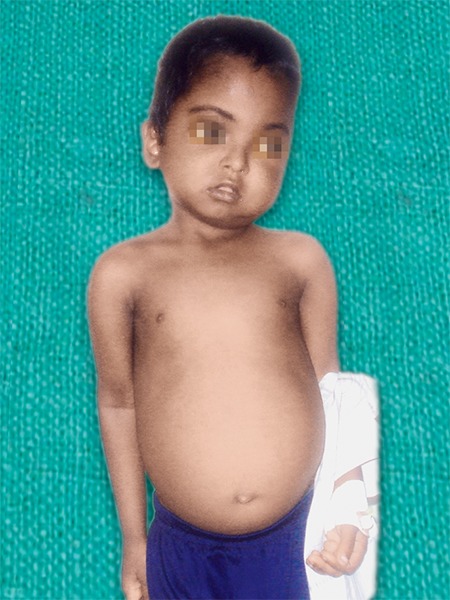
General appearance

**Fig. 2:**
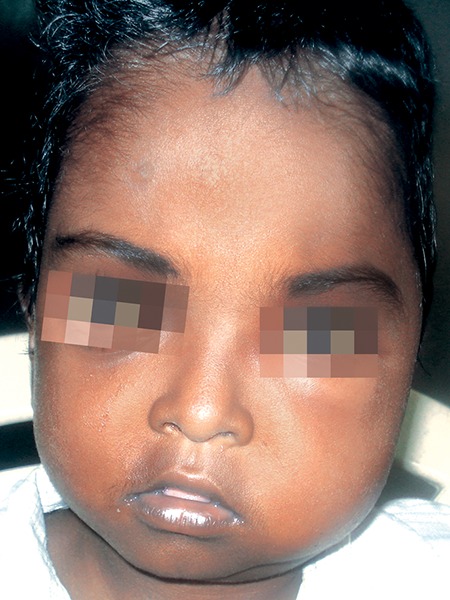
Facial photograph

On general examination, the patient was short stat-ured, poorly built and nourished but was conscious and co-operative. There was moderate pallor. Abdomen was distended with palpable liver and spleen. He was hypotensive and his pulse rate was irregular. Head and neck examination revealed brachycephalic skull with hypertelorism and orbital proptosis and microstomia. There was two significant enlarged tender left submandibular lymph nodes. On inspection, there was a diffuse swelling on the left side of middle part of the face over the left zygoma region. The skin over the swelling was stretched and shiny (Figs 1 and 2). On palpation, there was a diffuse tender soft swelling over the left zygoma region, lateral to the ala of the nose, 0.5 cm inferior to the left eye lid, anterior to the tragus of the ear, 2 cm superior to the lower border of the mandible, posterosuperior to the angle of the mouth involving the buccal, canine and infraorbital and temporal space. Intraoral examination revealed edentulous upper and lower arches with pus discharge from the posterior aspect of 65 region and from the buccal sulcus. There was also edema of the palate of varying consistency.

Laboratory investigations revealed a leukoeryhtroblastic blood picture. He had microcytic hypochromic anemia with ovalocytes and tear drop cells. WBC count was normal with a mild left shift and some reactive lymphocytes.

**Fig. 3:**
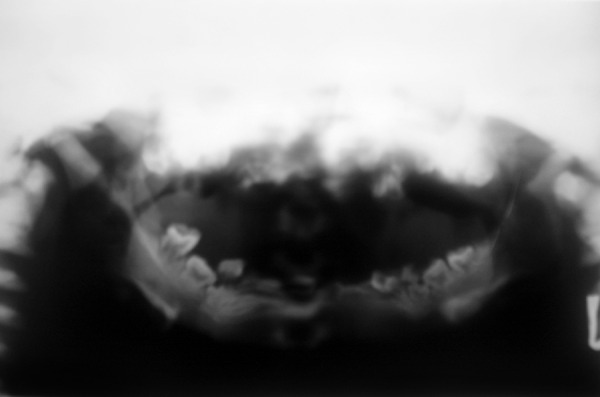
Panoramic radiographs showing indistinct head of condyle and coronoid process, with an increased gonial angle, diffuse radiopacity of the jaw

Radiographic investigation revealed diffuse sclerosis of bone. Chest radiograph showed increased bone density with clear lung fields. The panoramic view showed indistinct head of condyle and coronoid process with an increased gonial angle. The trabecular pattern and marrow spaces could not be appreciated clearly. There was a diffuse radiopacity of the jaw whereby the mandibular canal could not be traced. The morphology of the unerrupted teeth was altered with only the enamel capping remaining and a well appreciable large pulp chamber. Complete absence of the permanent tooth follicle was noticed (Fig. 3).

**Fig. 4:**
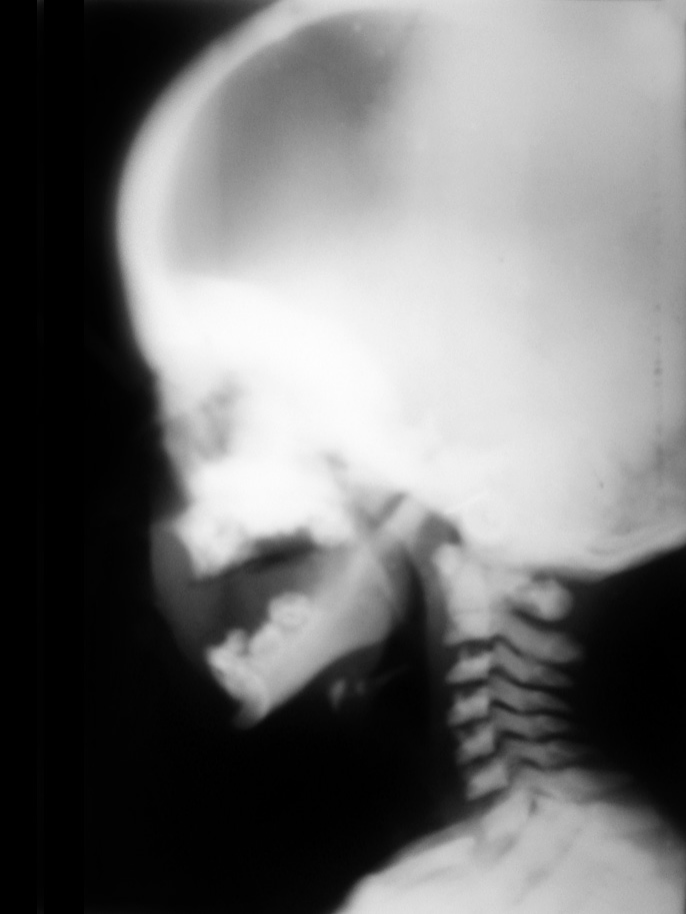
Lateral skull view showing increased radiopacity of the base of the skull, roof of the orbit and narrowing of the sella turcica. Increased radiopacity of maxilla and mandible with multiple unerrupted tooth buds. Note the typical “endobone” or bone within bone appearance in the cervical vertebrae.

Lateral skull view showed increased radiopacity of the base of the skull and roof of the orbit. There was narrowing of the sella turcica. The maxilla and mandible showed an increased radiopacity with multiple unerrupted tooth buds. Typical endobone or bone within bone appearance was noticed in the cervical vertebrae (Fig. 4).

**Fig. 5:**
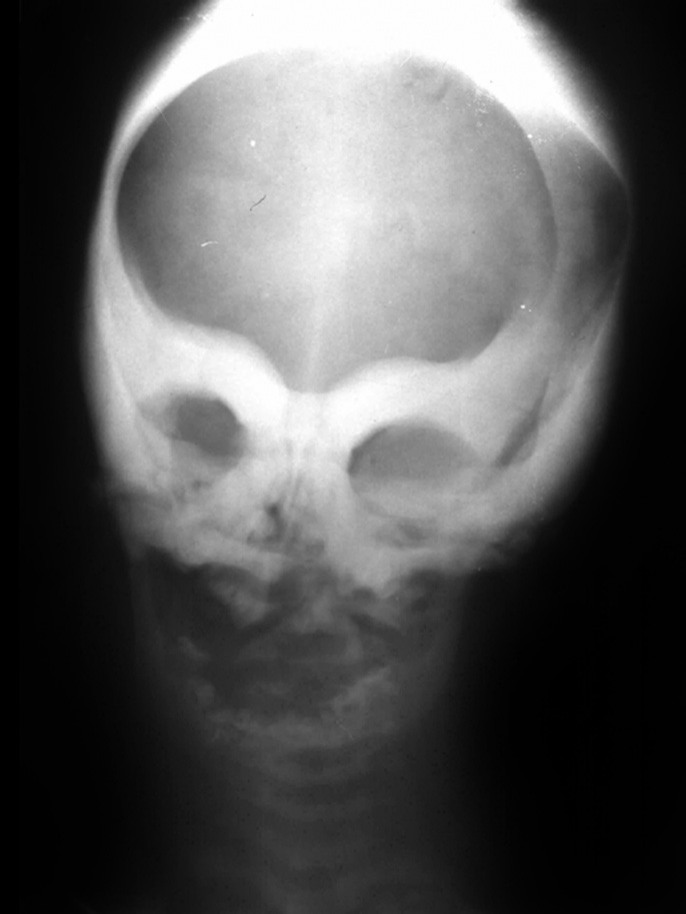
Posteroanterior view showing diffuse sclerosis of the skull, supraorbital ridge and body of the mandible and complete obliteration of the maxillary and frontal sinus

Posteroanterior view showed diffuse sclerosis of the skull, supraorbital ridge and body of the mandible. There was complete obliteration of the maxillary and frontal sinus (Fig. 5). Based on the clinical and radiographic findings, the patient was diagnosed as a case of osteomyelitis of the maxilla in infantile malignant osteopetrosis.

As the facility of hyperbaric oxygen therapy was not available in our set-up, the patient was not willing for further investigations and he was put on systemic antibiotic therapy and local debridement of the affected jaw was done.

## DISCUSSION

Osteopetrosis is a heritable bone disease showing genotypic and phenotypic variability and characterized by increased bone density. Albers-Schonberg first reported osteopetrosis in 1904. In 1921, Schulze described it as marmorknochen/ marble bone disease after observing this peculiar appearance in six cases from German literature. In 1926, Karshner termed this disease entity ‘osteopetrosis’ (stone-like or petrified bone) and noted that the increased hardness more closely resembled limestone than that of marble. Subsequently, many surgical reports described the consistency of osteopetrotic bone as chalk-like.^1^ It is otherwise called as Albers-Schonberg disease or marble bone disease and is characterized by a symptom complex related to altered bone metabolism. Overall incidence is estimated to be one case in 1,00,000-5,00,000 population. Three clinically distinct forms of osteopetrosis are recognized infantile malignant autosomal recessive form, fatal within few years of life (in the absence of effective therapy); intermediate autosomal recessive form appears during the first decade of life but does not follow the malignant course and the adult benign dominant form with full life expectancy but many orthopedic problems.^1,2^ Disease represents a spectrum of clinical variants because of the heterogeneity of genetic defects resulting in osteoclast dysfunction.

Malignant infantile osteopetrosis is a rare congenital disorder of bone resorption. It is caused by the failure of osteoclast to resorb immature bone. This leads to abnormal bone marrow cavity formation and clinically to the signs and symptoms of bone marrow failure. Pathologically, there is a persistance of primary spongiosa characterized by cores of calcified cartilage within the bone. Abnormal remodelling of primary woven bone to lamellar bone results in brittle bone that is prone to fracture. Multiple fractures, visual impairment and bone marrow failure are classic features of this disease.

The dental changes may vary from delayed eruption, early loss of teeth, missing teeth, malformed roots and crowns, teeth that are poorly calcified and prone to caries and thickened lamina dura. ^5^ There is reduced blood circulation due to obliteration and fibrosis of the marrow. Osteomyelitis secondary to carious exposure of dental pulps is a recognized complication in osteopetrosis and may contribute to early death of the patient. In infantile malignant OP, skeletal abnormalities are obvious at birth and symptoms from several organs present within weeks. Enlarged cranial bones give the head a characteristic appearance with a large rounded forehead. The eye sockets are shallow, making the eyes protrude. The nose is often flat, decreasing the size of the nasal cavity and leading to frequent congestion, as was seen in our patient. The head and body are unusually heavy, and balance problems may result. Sitting up is often difficult, and these children rarely learn to walk. The change in bone structure is accompanied by a marked tendency towards fragility and fractures may be sustained even in trivial accidents. As the blood vessels passing through the skull may also be compressed, more generalized neurological symptoms may develop, including developmental delay consistent with the features of our patient.^4,5^

As teeth develop within the defective bone tissue, both the primary and the permanent dentition are often affected. Most teeth fail to erupt, or tooth enamel may be of poor quality and vulnerable to caries.^4,5^ When abnormal bone growth compresses the marrow, pancytopenia develops. There is a high-risk of developing severe infections, like sepsis, and particularly osteomyelitis typically in mandible. The body to some extent compensates for bone marrow failure by extra-medullary hematopoiesis, resulting in hepatosplenomegaly as noted in the present case.^4,5^

Radiographs may show uniform increase in bone density without corticomedullary demarcation. The long bones have a dense chalk-like appearance. They may have an ‘Erlenmeyer flask’ deformity at their ends due to failure of mataphyseal remodeling, giving gross distal under tabulation and the presence of dense bone, vertical fine radiolucen-cies extending to the metaphysic are present probably due to vascular channels being better seen against dense bone. Fractures are usually transverse and heal with normal callus. The vertebral column has a ‘sandwich’ or ‘rugger jersey’ spine appearance with dense sclerotic bone at each end plate of the vertebral body. Spondylosis of the lumbar spine has been reported. The most common complication is pathologic fractures, those with congenital presentation are likely to have the most fractures. Femoral fractures are followed by posterior tibia. Upper extremity fractures are also reported. Nonunion and delayed union of fracture may occur.^6^

Management of the patients with this condition requires comprehensive approach to characteristic clinical problems, including hematological and metabolic abnormalities, fractures, deformity back pain, bone pain, osteomyelitis and neurological sequelae. Medical management of osteo-petrosis is based on efforts to stimulate host osteoclasts on provide in alternate source of osteoclasts. Stimulation of host osteoclasts has been attempted with calcium restriction, calcitriol, steroids, parathyroid hormone and inter-feron. Hyperbaric oxygen has been used in the treatment of mandibular osteomyelitis. Bone marrow transplant has been used with cure for infantile malignant osteopetrosis. If untreated, infantile osteopetrosis usually results in death by the first decade of life due to severe anemia, bleeding or infection. Osteomyelitis secondary to osteopetrosis tends to be refractory because of the reduced blood supply and accompanying anemia and neutropenia. Surgical resection should be planned with caution as osteopetrotic bone has less capacity to heal^6^ and these children are at risk of adverse respiratory events and increased perioperative morbidity and mortality as anesthetic complications.^1^

As osteopetrosis is likely to represents a spectrum of underlying etiologies resulting in osteoclast dysfunction, effective therapies need to be individualized.

## CONCLUSION

Infantile malignant OP is a lethal disease. The difficulty in obtaining conclusive results by sonograpic evaluation of fetus *in utero* makes prenatal molecular diagnosis highly desirable.^7^ Although diagnosis of infantile malignant osteopetrosis is easy and depends mainly on radiographic examination, it is often delayed due to rarity of the disease and lack of clinical suspicion. Osteomyelitis is a well-documented complication of osteopetrosis and is attributed to the adverse effects of osteopetrosis on local tissue vascular perfusion.
